# Changes in maternal hemoglobin during pregnancy and birth outcomes

**DOI:** 10.1186/s12884-015-0516-1

**Published:** 2015-04-02

**Authors:** Seung Chik Jwa, Takeo Fujiwara, Yuji Yamanobe, Kazuto Kozuka, Haruhiko Sago

**Affiliations:** Department of Social Medicine, National Research Institute for Child Health and Development, National Center for Child Health and Development, 2-10-1, Okura, Setagaya-ku, Tokyo 157-8535 Japan; Center of Maternal-Fetal, Neonatal and Reproductive Medicine, National Center for Child Health and Development, 2-10-1, Okura, Setagaya-ku, Tokyo 157-8535 Japan; Department of Information Technology and Management, National Center for Child Health and Development, 2-10-1, Okura, Setagaya-ku, Tokyo 157-8535 Japan

**Keywords:** Anemia, Birth weight, Hemoglobin, Placental weight, Pregnancy

## Abstract

**Background:**

The relationship between maternal hemoglobin (Hb) levels during pregnancy and birth outcomes has been controversial. Changes in Hb level during pregnancy may have an impact on birth outcomes. This study aimed to investigate whether changes in Hb levels from early to mid- or late pregnancy is associated with birth outcomes.

**Methods:**

Participants were singleton mothers who delivered at the National Center for Child Health and Development between 34 and 41 weeks of gestation in 2010 and 2011 (n = 1,986). Hb levels were measured at three time points: early (<16 weeks), mid- (16–27 weeks), and late (28–36 weeks) pregnancy. Associations between changes in Hb levels from early to mid- or late pregnancy and birth outcomes (birth weight, Z-score of birth weight, placental weight, and placental ratio) were assessed by multiple regression, adjusting for maternal and fetal covariates.

**Results:**

A smaller reduction in Hb levels from early to mid- or late pregnancy was significantly associated with lower birth weight, Z-score of birth weight, placental weight, and placental ratio. Compared to women with an intermediate reduction from early to late pregnancy, women with the least reduction had a significantly increased risk of delivering low birth weight (LBW) (adjusted odds ratio [aOR], 2.0; 95% confidence interval [CI], 1.3-3.1) and small-for-gestational-age (SGA) (aOR, 1.6; 95% CI, 1.04-2.3) infants, while women with the greatest reduction had a significantly decreased risk of delivering SGA (aOR, 0.38; 95% CI, 0.23-0.65) infants, but an increased risk of high placental ratio (aOR, 1.7; 95% CI, 1.2-2.5).

**Conclusions:**

Hb changes from early to mid- or late pregnancy were inversely associated with birth weight, placental weight, and placental ratio.

## Background

The relationship between maternal hemoglobin (Hb) levels during pregnancy and adverse birth outcomes has been a source of controversy. Several studies have reported that severe anemia during “early” pregnancy is associated with adverse birth outcomes, such as low birth weight (LBW) [1-4], although other studies have showed no such association [5-8]. It has also been reported that Hb levels at “mid- or late” pregnancy are inversely associated with birth weight and placental weight [3,6,9,10]. This suggests that the association between Hb levels during pregnancy and birth outcomes may differ based on the stage of pregnancy assessed.

Physiologically, plasma volume increases by 10 to 15 percent at 6 to 12 weeks of gestation and increases rapidly thereafter until 30 to 34 weeks [11,12]. This induces a modest decrease in Hb levels during pregnancy. Even in healthy pregnant women, the lack of an increase in plasma volume during pregnancy is associated with lower birth weight and placental weight, and an increased risk of delivering a small-for-gestational-age (SGA) infant [12-14]. However, the impact of changes in Hb levels from early to mid- or late pregnancy on birth outcomes has not been investigated in detail.

We hypothesized that a reduction in Hb levels from early pregnancy to mid- or late pregnancy is a proxy for plasma volume increase during pregnancy and thus protects against adverse birth outcomes including LBW, SGA, and high placental ratio. Thus, the current study aims to investigate the impact of changes in Hb levels from early to mid- or late pregnancy on birth outcomes, including birth weight, placental weight, and placental ratio.

## Methods

### Study design and sample population

This is a secondary analysis using records from a hospital electronic health record system of mothers who delivered at the National Center for Child Health and Development (NCCHD, Tokyo, Japan) between January 2010 and December 2011. We used information collected for routine antenatal care. Written informed consent for data use is obtained from patients routinely at the first antenatal visit. The institutional review board at the NCCHD approved this study (institutional review board approval no. 34). Expected delivery dates (i.e., gestational age) were routinely confirmed by an obstetrician via ultrasound.

**Figure 1:**
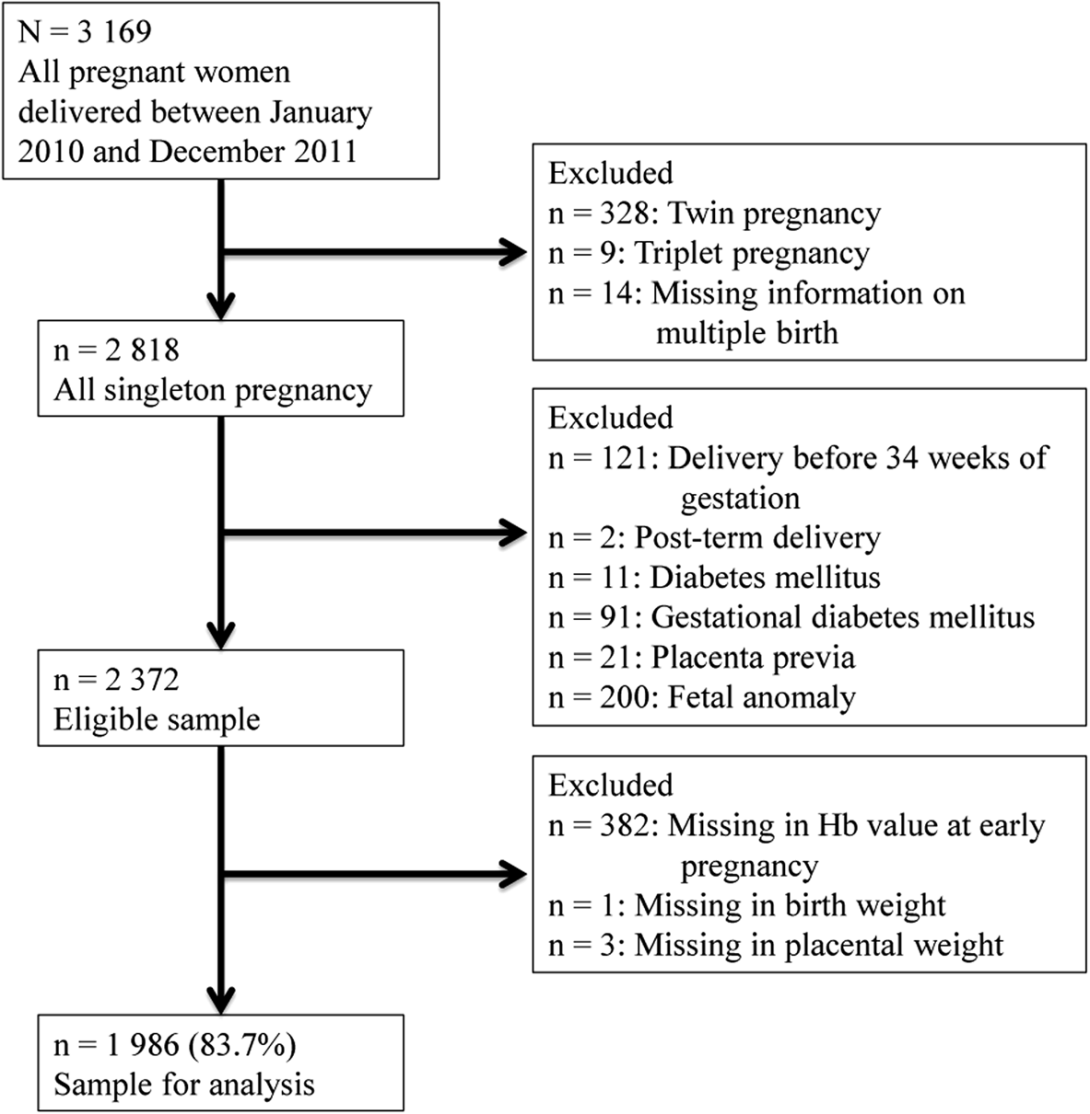
**Participant flow chart.**

Inclusion criteria were: 1) singleton pregnancy, 2) Hb measured at <16 weeks of gestation, and 3) participants who delivered at the NCCHD between 34 and 41 weeks of gestation. We excluded early preterm births (i.e., <34 weeks of gestation) because these cases mostly lacked Hb measurements in late pregnancy. We also excluded subjects with pre-pregnancy diabetes mellitus (DM), gestational diabetes mellitus (GDM), placenta previa, and fetal anomalies. We did not exclude cases with hemoglobinopathy, including the thalassemia trait, since prevalence of hemoglobinopathy in Japan is quite low ranging from 1 per 2,500 to 1 per 4,000 [15]. A participant flow chart is shown in Figure 1. A total of 3,169 women delivered at the NCCHD during the study period. Of these, the following were excluded: 328 twin births, 9 triplet births, 14 with missing information for multiple births, 11 with pre-pregnancy DM, 91 with GDM, 50 with placenta previa, and 200 with fetal anomalies. In addition, 121 deliveries before 34 weeks of gestation, 2 post-term deliveries, 354 with missing Hb values at early pregnancy, 1 with missing birth weight, and 2 with missing placental weight were excluded. Thus, a total of 1,986 pregnancies were included in the analysis.

### Hb measurements

Three Hb measurements were performed for routine antenatal care. The first sample was collected before 16 weeks of gestation (early Hb), the second between 16 weeks 0 days and 27 weeks 6 days gestation (mid-Hb), and the third after 28 weeks 0 days gestation (late Hb). Hb levels were measured with a calibrated laboratory machine (ADVIA 2120 hematology system, SIEMENS health care) immediately after blood sample collection. Anemia was defined as Hb <11.0 g/dL, according to the World Health Organization definition [16]. Hb changes from early to mid- or late pregnancy (ΔHbs) were calculated as mid-Hb minus early Hb, and late Hb minus early Hb, respectively, and stratified by tertiles based on the sample distribution.

### Birth outcomes

Primary outcomes were birth weight, Z-score of birth weight, placental weight, and placental ratio. The placental ratio was calculated as placental weight (g) divided by birth weight (g). The Z-score of birth weight was calculated from the Japanese national reference for birth weight [17]. For further analysis, continuous outcomes were dichotomized as follows: LBW, SGA, and high placental ratio. LBW was defined as being below 2,500 g and SGA was defined as being below the 10^th^ percentile of the national reference. High placental ratio was defined as a placental ratio above the 90^th^ percentile of the sample.

Following deliveries (both vaginal and cesarean section), the umbilical cord was clamped and cut about 7–8 cm from the side of the infant. After removing clots, the untrimmed placenta and umbilical cord were weighed by midwives and recorded in electronic format. Although the use of the untrimmed placenta and umbilical cord may introduce some bias into the placental weight measurements, we considered this method consistent with another study and believe any effects would be negated by the large sample size [18]. Placental weight did not differ by delivery method. The weights of all infants were measured immediately after delivery upon completion of a physical examination. Pregnancy complications and delivery summaries were also recorded in electronic format by obstetricians.

### Covariates

Information on participant age, pre-pregnancy body weight, height, parity, artificial reproductive technology (ART) for conception, past medical complications (i.e., hypertension, autoimmune disease, Basedow’s disease (i.e. hyperthyroidism), and hypothyroid disease), and smoking and drinking status were collected by questionnaire at the time of the first antenatal visit and recorded in the NCCHD medical database. Pre-pregnancy BMI was calculated as weight [kg]/height^2^ [m]. Data regarding iron prescriptions, blood pressure (BP) before 16 weeks of gestation, and body weight at the last antenatal visit were also retrieved from the database. Participant weight gain during pregnancy was calculated as body weight at the last antenatal visit [kg] before delivery minus pre-pregnancy body weight [kg]. BP measured before 16 weeks of gestation at the antenatal visit was converted into mean arterial pressure (MAP) as follows: MAP = (2*diastolic BP + systolic BP)/3. Fetal sex was also retrieved from the delivery summary.

### Statistical analysis

To evaluate the direct effect of Hb levels at different gestational ages and Hb changes from early pregnancy on birth weight, Z-score of birth weight, placental weight, and placental ratio, we conducted multiple linear regression analysis, adjusting for maternal and fetal covariates. We first analyzed the association between Hb levels at early, mid-, and late pregnancy and birth outcomes without adjustment, and defined this as the crude model. We then adjusted for potential confounders including maternal age, parity, pre-pregnancy BMI, smoking status, ART, and fetal sex, and defined this as Model 1. We further adjusted for MAP at early pregnancy and past medical complications (i.e., hypertension, autoimmune disease, Basedow’s disease, and hypothyroid disease), and defined this as Model 2. As with the analysis of Hb levels and birth outcomes, we evaluated the association between Hb changes from early to mid- or late pregnancy and birth outcomes by replacing Hb levels with Hb changes from early pregnancy in the previous models. We also added Hb levels at early pregnancy to Model 2 and defined this as Model 3 in order to evaluate the independent effect of changes of Hb levels from early pregnancy on birth outcomes. The placental ratio was multiplied by 1000 for regression analysis since the co-efficient of each independent variable was too small. Finally, we evaluated risks of LBW, SGA, and high placental ratio according to tertiles of changes in Hb levels from early to mid- or late pregnancy by multiple logistic regression analysis. Trends across tertiles of changes in Hb levels from early pregnancy were tested by treating the level of exposure as an ordinal variable in the logistic model.

Since there were missing values for mid- (5.4%) and late (0.8%) Hb and several other covariates from 1.2% in pre-pregnancy BMI to 9.6% in MAP, multiple imputation was performed. Model-wise deletion was also performed as sensitivity analysis and the results did not change. P < 0.05 was considered statistically significant. All analyses were performed with Stata SE software (Version 12; Stata Corporation, College Station, TX).

## Results

Baseline characteristics of the sample population stratified by tertiles of Hb changes from early to late pregnancy are shown in Table 1. The mean maternal age was 35.5 years (standard deviation [SD] = 4.2), 53% of participants were nulliparous, and 16% used ART for conception. Women who were classified as having the least reduction from early to late pregnancy (i.e. –1.0 ≤ Hb change [g/dL]) were more likely to be nulliparous, and had lower pre-pregnancy BMI and MAP at early pregnancy.

**Table 1:** **Baseline characteristics at early pregnancy stratified by tertiles of Hb changes from early to late pregnancy (n = 1986)** ^**a, b**^

**Characteristics**	**Total (n = 1986)**	**Greatest reduction (Hb change < −1.9) (n = 639)**	**Intermediate reduction (−1.9 ≤ Hb change < −1.0) (n = 689)**	**Least reduction (Hb change ≥ −1.0) (n = 658)**
Maternal age, (years)	35.5 (4.2)	35.6 (4.1)	35.4 (4.2)	35.4 (4.4)
Pre-pregnancy BMI, (kg/m^2^)	20.3 (2.6)	20.5 (2.7)	20.3 (2.5)	20.0 (2.6)
Maternal weight gain until delivery, (kg)	9.6 (3.4)	9.5 (3.4)	9.7 (3.5)	9.6 (3.2)
Parity				
0, n (%)	1057 (53.2)	296 (46.3)	373 (54.1)	388 (59.0)
1, n (%)	730 (36.8)	273 (42.7)	251 (36.4)	206 (31.3)
≥2, n (%)	199 (10.0)	70 (11.0)	65 (9.4)	64 (9.7)
ART, n (%)	310 (15.6)	101 (15.8)	108 (15.7)	101 (15.3)
Maternal pre-pregnancy complications				
Hypertension, n (%)	25 (1.3)	11 (1.7)	9 (1.3)	5 (0.76)
Autoimmune disease, n (%)	44 (2.2)	14 (2.2)	15 (2.2)	15 (2.3)
Basedow’s disease, n (%)	28 (1.4)	10 (1.6)	13 (1.9)	5 (0.76)
Hypothyroid, n (%)	51 (2.6)	13 (2.0)	22 (3.2)	16 (2.4)
Smoking				
Never or former, n (%)	1981 (99.7)	638 (99.8)	688 (99.9)	655 (99.5)
Current, n (%)	5 (0.25)	1 (0.16)	1 (0.15)	3 (0.46)
Alcohol intake				
Current, n (%)	158 (8.0)	48 (7.5)	58 (8.4)	52 (7.9)
MAP at early pregnancy, (mmHg)^c^	79.5 (9.6)	80.8 (9.7)	79.5 (9.6)	78.2 (9.4)

^a^Values are presented as mean (SD) for continuous variables and n (%) for dichotomous variables.

^b^Hb tertiles were calculated from the distribution of Hb changes from early to late pregnancy (i.e., Hb at late pregnancy - Hb at early pregnancy).

^c^Calculated as (2 x diastolic BP + systolic BP)/3.

ART, artificial reproductive technology; MAP, mean arterial pressure; SD, standard deviation.

Hb levels during pregnancy and birth outcomes stratified by tertiles of Hb changes from early to late pregnancy are shown in Table 2. Mean gestational ages at the time of Hb measurements (weeks) were 11.4 (SD = 2.0) at early pregnancy, 24.7 (SD = 1.5) at mid-pregnancy and 35.4 (SD = 1.3) at late pregnancy. Mean Hb levels were 12.5 g/dL (SD = 0.9) at early pregnancy, 11.0 g/dL (SD = 0.9) at mid-pregnancy and 11.1 g/dL (SD = 1.0) at late pregnancy. Ninety-one participants (4.5%) were anemic at early pregnancy, and this dramatically increased to 876 (44.1%) in mid-pregnancy and 907 (45.7%) in late pregnancy. Women with the least reduction from early to late pregnancy were more likely to be anemic during early pregnancy, but less likely during late pregnancy. Iron supplementation was prescribed more often to women with the least reduction at mid-pregnancy, but less likely in late pregnancy.

**Table 2:** **Hb levels at early, mid-, and late pregnancy and birth outcomes stratified by tertiles of Hb changes from early to late pregnancy (n = 1986)** ^**a, b**^

**Variable**	**Total (n = 1986)**	**Greatest reduction (Hb change < −1.9) (n = 639)**	**Intermediate reduction (−1.9 ≤ Hb change < −1.0) (n = 689)**	**Least reduction (Hb change ≥ −1.0) (n = 658)**
**Early pregnancy (<16 weeks)**				
Hb, (g/dL)	12.5 (0.88)	13.0 (0.73)	12.5 (0.75)	12.0 (0.87)
Anemia (<11 g/dL), n (%)	89 (4.5)	4 (0.63)	14 (2.0)	70 (10.6)
Gestational age at measurement, (weeks)	11.4 (2.0)	11.0 (1.9)	11.4 (1.9)	11.7 (2.0)
Iron supplement intake	12 (0.60)	0 (0)	1 (0.15)	11 (1.7)
**Mid-pregnancy (16–27 weeks)**				
Hb, (g/dL)	11.0 (0.86)	11.1 (0.79)	11.1 (0.83)	11.0 (0.95)
Anemia (<11 g/dL), n (%)	876 (44.1)	276 (43.2)	288 (41.8)	312 (47.4)
Gestational age at measurement, (weeks)	24.7 (1.5)	24.6 (1.5)	24.7 (1.6)	24.8 (1.5)
Iron supplement intake	290 (14.6)	59 (9.2)	79 (11.5)	152 (23.1)
**Late pregnancy (28–37 weeks)**				
Hb, (g/dL)	11.1 (0.96)	10.4 (0.79)	11.0 (0.77)	11.7 (0.84)
Anemia (<11 g/dL), n (%)	907 (45.7)	473 (74.0)	316 (45.9)	118 (17.9)
Gestational age at measurement, (weeks)	35.4 (1.3)	35.3 (1.4)	35.4 (1.3)	35.5 (1.2)
Iron supplement intake	408 (20.5)	228 (35.7)	102 (14.8)	78 (11.9)
**Pregnancy complication**				
Pregnancy-induced hypertension, n (%)	44 (2.2)	15 (2.3)	14 (2.0)	15 (2.3)
**Delivery outcomes**				
Gestational age of delivery, (week)	39.1 (1.3)	39.1 (1.3)	39.3 (1.2)	39.3 (1.3)
Preterm birth, n (%)	68 (3.4)	24 (3.8)	22 (3.2)	22 (3.3)
Mode of delivery				
Normal vaginal delivery, n (%)	1205 (60.7)	367 (57.4)	422 (61.2)	416 (63.2)
Forceps or vacuum delivery, n (%)	297 (15.0)	97 (15.2)	105 (15.2)	95 (14.4)
Cesarean section, n (%)	484 (24.4)	175 (27.4)	162 (23.5)	147 (22.3)
Sex (male), n (%)	1029 (51.8)	322 (50.4)	363 (52.7)	344 (52.3)
Birth weight, (g)	3016 (370)	3068 (354)	3032 (369)	2947 (386)
Z-score of birth weight	0.078 (0.94)	0.27 (0.89)	0.096 (0.93)	−0.13 (0.95)
Low birth weight, n (%)	132 (6.6)	32 (5.0)	38 (5.5)	62 (9.4)
Small for gestational age, n (%)^c^	139 (7.0)	22 (3.4)	51 (7.4)	66 (10.0)
Placental weight, (g)	546 (101)	572 (101)	545 (100)	523 (98)
Placental ratio	0.18 (0.027)	0.19 (0.029)	0.18 (0.026)	0.18 (0.026)
High placental ratio, n (%)^d^	192 (9.7)	87(13.6)	60 (8.7)	45 (6.8)

^a^Values are presented as mean (SD) for continuous variables and n (%) for dichotomous variables.

^b^Hb tertiles were calculated from the distribution of Hb changes from early to late pregnancy (i.e., Hb at late pregnancy - Hb at early pregnancy).

^c^SGA is defined as being below the 10^th^ percentile of the Japanese national reference.

^d^High placental ratio is defined as being over the 90^th^ percentile of the sample population.

Mean gestational age at delivery was 39.1 weeks (SD = 1.3). In the study population, 1,205 women (60.7%) underwent vaginal deliveries, 297 women (15.0%) had forceps or vacuum deliveries, and 484 women (24.4%) had Cesarean deliveries. Women with the greatest reduction from early to late pregnancy had a higher rate of Cesarean deliveries (27.4%) compared with women with the least reduction (22.3%). Mean birth weight was 3,016 g (SD = 370), mean placental weight was 546 g (SD = 101), and mean placental ratio was 0.18 (SD = 0.027). There were 132 (6.2%) LBW and 139 (7.0%) SGA infants. Women with the least reduction from early to late pregnancy tended to have a lower birth weight, Z-score of birth weight, and placental weight, resulting in a higher proportion of LBW and SGA infants compared with women in the intermediate or greatest reduction groups.

The results of multiple regression analysis for the association between Hb levels at early, mid-, or late pregnancy and birth outcomes are shown in Table 3. Hb levels at early pregnancy were not significantly associated with birth weight, Z-score of birth weight, placental weight, and placental ratio, whereas Hb levels at mid- and late pregnancy were significantly inversely associated with these outcomes. This suggests that if Hb levels increased +1 g/dL at mid- or late pregnancy, birth weight changed by −57.9 g (95% CI, −77.3 to −38.5) and −73.2 g (95% CI, −90.0 to −56.4), respectively, in the adjusted model. Similar significant inverse associations were observed for Z-score of birth weight, placental weight, and placental ratio.

**Table 3:** **Association between Hb levels at early, mid-, and late pregnancy and birth outcomes (n = 1986)**

**Birth outcome**	**Hb levels during pregnancy**	**Crude model**		**Model 1**		**Model 2**	
		**Coefficient**	**95% CI**	**Coefficient**	**95% CI**	**Coefficient**	**95% CI**
Birth weight (g)	Early pregnancy	−13.6	−32.0 to 4.8	−25.1*	−43.5 to −6.7	−18.1	−36.8 to 0.49
	Mid-pregnancy	−55.3**	−74.7 to −35.9	−63.4**	−82.6 to −44.2	−57.9**	−77.3 to −38.5
	Late pregnancy	−71.0**	−88.0 to −53.9	−75.9**	−92.8 to −59.1	−73.2**	−90.0 to −56.4
Z-score of birth weight	Early pregnancy	−0.0035	−0.050 to 0.043	−0.039	−0.086 to 0.0074	−0.028	−0.076 to 0.019
	Mid-pregnancy	−0.14**	−0.19 to −0.088	−0.16**	−0.21 to −0.11	−0.15**	−0.20 to −0.10
	Late pregnancy	−0.19**	−0.23 to −0.14	−0.20**	−0.24 to −0.16	−0.20**	−0.24 to −0.16
Placental weight (g)	Early pregnancy	2.1	−2.9 to 7.1	−1.0	−6.1 to 4.0	−0.55	−5.7 to 4.6
	Mid-pregnancy	−15.5**	−20.8 to −10.2	−17.7**	−23.0 to −12.4	−17.6**	−23.0 to −12.3
	Late pregnancy	−22.4**	−27.0 to −17.9	−23.3**	−27.9 to −18.7	−23.3**	−27.9 to −18.8
Placental ratio (x1000)	Early pregnancy	1.55*	0.20 to 2.9	1.2	−0.18 to 2.5	0.91	−0.48 to 2.3
	Mid-pregnancy	−1.8*	−3.2 to −0.35	−2.1*	−3.5 to −0.63	−2.4*	−3.9 to −0.93
	Late pregnancy	−3.1**	−4.4 to −1.9	−3.1**	−4.4 to −1.9	−3.3**	−4.6 to −2.0

*P < 0.05, **P < 0.001.

Model 1: adjusted for maternal age, parity, pre-pregnancy BMI, smoking, ART, fetal sex.

Model 2: adjusted for Model 1 + MAP at early pregnancy and medical complications (hypertension, autoimmune disease, Basedow’s disease, hypothyroid disease).

ART, artificial reproductive technology; CI, confidence interval; Hb, Hemoglobin; MAP, mean arterial pressure; Z-score, z-score of birth weight.

The effects of Hb changes from early pregnancy to mid- or late pregnancy on birth outcomes are shown in Table 4. Hb changes from early to mid- or late pregnancy were significantly inversely associated with birth weight, Z-score of birth weight, placental weight, and placental ratio, after adjusting for confounders and Hb levels in early pregnancy. The results indicate that a +1 g increase in Hb from early to late pregnancy was associated with a −76.1 (95% CI, −94.0 to −58.2) change in birth weight (g), a −0.21 (95% CI, −0.26 to −0.17) change in Z-score of birth weight, a −26.1 (95% CI, −30.9 to −21.2) change in placental weight (g), and a −4.0 (95% CI, −5.4 to −2.7) change in placental ratio (x1000), in the adjusted model.

**Table 4:** **Association between Hb changes (ΔHb) from early to mid- or late pregnancy and birth outcomes (n = 1986)**

**Birth outcome**	**Hb changes from early pregnancy**	**Crude model**		**Model 1**		**Model 2**		**Model 3**	
		**Coefficient**	**95% CI**	**Coefficient**	**95% CI**	**Coefficient**	**95% CI**	**Coefficient**	**95% CI**
Birth weight (g)	ΔHb (mid-Hb – early Hb)	−51.0**	−72.9 to −29.1	−45.2**	−67.0 to −23.4	−46.9**	−68.5 to −25.2	−73.5**	−98.0 to −48.9
	ΔHb (late Hb – early Hb)	−48.8**	−64.4 to −33.3	−45.0**	−60.4 to −29.5	−47.8**	−63.1 to −32.4	−76.1**	−94.0 to −58.2
Z-score of birth weight	ΔHb (mid-Hb – early Hb)	−0.17**	−0.22 to −0.11	−0.15**	−0.20 to −0.093	−0.15**	−0.21 to −0.096	−0.21**	−0.28 to −0.15
	ΔHb (late Hb – early Hb)	−0.15**	−0.19 to −0.11	−0.14**	−0.18 to −0.10	−0.14**	−0.18 to −0.11	−0.21**	−0.26 to −0.17
Placental weight (g)	ΔHb (mid-Hb – early Hb)	−22.5**	−28.4 to −16.5	−20.7**	−26.7 to −14.8	−20.8**	−26.7 to −14.9	−27.3**	−34.0 to −20.6
	ΔHb (late Hb – early Hb)	−20.0**	−24.2 to −15.8	−18.4**	−22.6 to −14.3	−18.8**	−22.9 to −14.6	−26.1**	−30.9 to −21.2
Placental ratio (x1000)	ΔHb (mid-Hb – early Hb)	−4.4**	−6.0 to −2.8	−4.2**	−5.8 to −2.6	−4.1**	−5.7 to −2.5	−4.7**	−6.5 to −2.8
	ΔHb (late Hb – early Hb)	−3.7**	−4.8 to −2.6	−3.4**	−4.5 to −2.2	−3.3**	−4.5 to −2.2	−4.0**	−5.4 to −2.7

**P < 0.001.

Model 1: adjusted for maternal age, parity, pre-pregnancy BMI, smoking, ART, fetal sex.

Model 2: adjusted for Model 1 + MAP at early pregnancy and medical complications (hypertension, autoimmune disease, Basedow’s disease, hypothyroid disease).

Model 3: adjusted for Model 3 + Hb levels at early pregnancy.

ART, artificial reproductive technology; CI, confidence interval; Hb, Hemoglobin; MAP, mean arterial pressure.

Results of logistic regression of Hb changes from early to mid- or late pregnancy for the outcomes of LBW, SGA, and high placental ratio are shown in Table 5. Women with the least reduction from early to mid-pregnancy (i.e. –1.0 ≤ Hb change) had a significantly increased risk of delivering SGA infants (adjusted OR [aOR], 1.6; 95% CI, 1.1 to 2.5) and a decreased risk of having a high placental ratio (aOR, 0.59; 95% CI, 0.39 to 0.90), compared with women with an intermediate reduction (i.e. –1.9 ≤ Hb change < −1.0). With respect to the analysis of Hb changes from early to late pregnancy, compared with women with an intermediate reduction, women with the least reduction had a significantly increased risk of delivering a LBW (aOR, 2.0; 95% CI, 1.3 to 3.1) or SGA (aOR, 1.6; 95% CI, 1.04 to 2.3) infant. On the other hand, women with the greatest reduction (i.e. Hb change < −1.9) had a significantly reduced risk of delivering a SGA (aOR, 0.38; 95% CI, 0.23 to 0.65) infant, and an increased risk of high placental ratio (aOR, 1.7; 95% CI, 1.2 to 2.5). This resulted in a significant linear trend between Hb tertiles from early to late pregnancy, and risks of LBW, SGA, and high placental ratio (p value for trend <0.001).

**Table 5:** **Adjusted OR and 95% CI of Hb changes from early pregnancy for low birth weight, small for gestational age, and high placental ratio** ^**a**^

	**LBW**	**SGA** ^**b**^	**High placental ratio** ^**c**^
	**Adjusted OR (95% CI)**	**Adjusted OR (95% CI)**	**Adjusted OR (95% CI)**
**Hb changes from early to mid-pregnancy** ^**d**^			
Greatest reduction (Hb change < −1.7) (n = 675)	1.2 (0.74 to 1.9)	0.72 (0.44 to 1.2)	1.1 (0.74 to 1.5)
Intermediate reduction (−1.7 ≤ Hb change < −1.1) (n = 680)	Reference	Reference	Reference
Least reduction (Hb change ≥ −1.1) (n = 631)	1.3 (0.84 to 2.1)	1.6 (1.1 to 2.5)	0.59 (0.39 to 0.90)
P for trend	0.66	0.001	0.02
**Hb changes from early to late pregnancy** ^**d**^			
Greatest reduction (Hb change < −1.9) (n = 639)	0.77 (0.46 to 1.3)	0.38 (0.23 to 0.65)	1.7 (1.2 to 2.5)
Intermediate reduction (−1.9 ≤ Hb change < −1.0) (n = 689)	Reference	Reference	Reference
Least reduction (Hb change ≥ −1.0) (n = 658)	2.0 (1.3 to 3.1)	1.6 (1.04 to 2.3)	0.76 (0.49 to 1.2)
P for trend	<0.001	<0.001	<0.001

^a^Adjusted for maternal age, parity, pre-pregnancy BMI, smoking, ART, fetal sex, MAP at early pregnancy, medical complications (hypertension, autoimmune disease, Basedow’s disease, hypothyroid disease), and Hb levels at early pregnancy.

^b^SGA is defined as being below the 10^th^ percentile of the Japanese national reference.

^c^High placental ratio is defined as being over the 90^th^ percentile of the sample population.

^d^Hb changes were calculated as Hb at mid- or late pregnancy – Hb at early pregnancy.

ART, artificial reproductive technology; CI, confidence interval; Hb, hemoglobin; LBW, low birth weight; MAP, mean arterial pressure; OR, odds ratio; SGA, small for gestational age.

## Discussion

In this study, we found that changes of Hb levels from early pregnancy were significantly inversely associated with birth weight, Z-score of birth weight, placental weight, and placental ratio in pregnant Japanese women. Women with the least reduction from early to late pregnancy had a significantly increased risk of delivering a LBW or SGA infant and a decreased risk of high placental ratio, compared to women with the intermediate or greatest reduction.

Although some studies have assessed the association of Hb levels at early [6,5,8,19,20], mid- [6,19,21,22], and late pregnancy [21,23,24], or minimum Hb levels during pregnancy [12,19,25], with LBW, SGA, and placental ratio, only a few studies have reported on the association between Hb changes during pregnancy and these birth outcomes [4,10,12]. Whittaker et al. studied serial hematologic changes, including Hb and plasma volume, throughout pregnancy in 69 women in the United Kingdom and reported no significant association between Hb changes from pre-pregnancy to late pregnancy and birth weight or placental weight, although Hb changes were negatively correlated with birth weight and placental weight [12]. However, their small sample size may not have had sufficient statistical power to detect the association between Hb changes and birth outcomes.

Bakacak et al. also investigated Hb levels at different trimesters in 329 pregnant Turkish women without maternal complications [4]. They demonstrated that women who delivered LBW infants had a smaller Hb reduction (ΔHb = −0.6, SD = 2.9) from early to late pregnancy compared with women who delivered babies weighing more than 3500 g (ΔHb = −1.5, SD = 3.6); however, the relationship did not reach a statistically significant level (P value = 0.06). Gonzales et al. also found that pregnant Peruvian women who were initially nonanemic (11–14.5 g/dL) at first booking and who later showed increased Hb (>14.5 g/dL) due to erythrocytosis in a second Hb measurement had a significant increased risk for SGA (aOR 1.66; 95% CI, 1.51 to 1.83) [10]. In our study, mothers with Hb values of more than 13.3 or 15.5 g/dL at late pregnancy were prone to have SGA or LBW infants respectively, regardless of iron supplementation (data not shown). Thus, women with Hb values in this range or higher should be closely monitored during pregnancy. In addition to those previous findings, we confirmed the significant inverse relationship between changes in Hb during pregnancy and birth weight in a Japanese population, and further found inverse associations between changes in Hb during pregnancy with placental weight, and placental ratio.

One potential explanation for the association between a reduction in Hb levels from early pregnancy and a larger birth weight is that Hb changes during pregnancy may reflect plasma volume expansion. A smaller reduction in Hb levels from early pregnancy may suggest a failure to expand plasma volume during pregnancy, which may impair fetoplacental circulation [26]. Whittaker et al. reported that changes in Hb levels from pre-pregnancy to late pregnancy were significantly correlated with changes in plasma volume [12], and reduced plasma volume expansion during pregnancy reportedly increases the risk of smaller birth weight and SGA [12,13].

We also found that Hb changes were significantly inversely associated with placental ratio, which may explain conflicting results from previous studies regarding anemia during pregnancy and the increased risk of high placental ratio. While one study found no association between Hb levels at the first antenatal visit and placental ratio [5], other studies have reported that Hb at later gestational ages, including the lowest level of Hb during pregnancy, were inversely associated with placental ratio [21,27,28]. Our findings suggest that changes in Hb levels from early to mid- or late pregnancy may have an independent effect on placental ratio regardless of Hb levels at early pregnancy, with a larger reduction in Hb levels from early pregnancy resulting in lower Hb levels at mid- or late pregnancy and an increased risk of high placental ratio. Given the increase in the number of reports suggesting an association between high placental ratios and adverse short- and long-term health outcomes [25,29-33], larger changes in Hb levels from early pregnancy may not necessarily reflect a favorable intrauterine environment.

This study has several limitations. First, the study was conducted in a single perinatal center where pregnant women with complications tend to deliver. The mean maternal age was higher than that of the general population in Japan (our study vs. general population, 35.5 vs. 30) [34]. Also, the proportion of women who conceived via ART was relatively high (16%). Thus, caution should be exercised when generalizing our results. However, routine antenatal care including the timing of Hb measurement during pregnancy and obstetric intervention such as the decision to terminate the pregnancy was consistent as the study was based at a single institution, and so differential measurement errors are less likely to have been introduced. Since delivery records were inputted directly by obstetricians to prevent misclassification in the database, critical misclassification is also less likely. Second, we did not evaluate the effect of iron intake during pregnancy. Hb changes from early pregnancy may, in part, be mediated by iron intake during pregnancy, including iron supplementation. Iron intake during pregnancy could account for a smaller reduction in Hb. However, we did not collect data regarding iron intake other than prescription information, and data on adherence to prescribed iron supplements and other non-prescribed iron supplements were not available for this study. Thus, randomized controlled trials of iron supplements are needed to evaluate the exact effect of iron supplements on Hb changes during pregnancy and on birth outcomes. Interestingly, two randomized controlled trials of routine prenatal iron supplement intake in initially non-anemic women from early to mid-pregnancy in the United States did not alter third trimester Hb levels, but significantly increased birth weight compared with the placebo group [35,36]. Thus, it is less likely that iron intake during pregnancy could cause a lower birth weight via a smaller reduction in Hb during pregnancy.

## Conclusions

Changes in Hb levels from early pregnancy were significantly inversely associated with birth weight, placental weight, and placental ratio. Although the underlying mechanisms are unknown, these results suggest that, in addition to Hb levels alone, changes in Hb levels from early pregnancy may impact birth outcomes. Given the findings of our study, further studies are needed to evaluate changes in Hb levels together with plasma volume during pregnancy and on birth outcomes in a more diverse population, with additional information on co-morbidities and iron supplementation during pregnancy. Importantly, our study suggests that clinicians may also consider changes in Hb levels in antenatal care in addition to Hb levels at a specific point in pregnancy.

## Abbreviations

ART: Artificial reproductive technology
aOR: Adjusted odds ratio
DM: Diabetes mellitus
GDM: Gestational diabetes mellitus
Hb: Hemoglobin
LBW: Low birth weight
MAP: Mean arterial pressure
SGA: Small for gestational age
